# Examining gray matter structure associated with academic performance in a large sample of Chinese high school students

**DOI:** 10.1038/s41598-017-00677-9

**Published:** 2017-04-18

**Authors:** Song Wang, Ming Zhou, Taolin Chen, Xun Yang, Guangxiang Chen, Meiyun Wang, Qiyong Gong

**Affiliations:** 1 Huaxi MR Research Center (HMRRC), Department of Radiology, West China Hospital of Sichuan University, Chengdu, 610041 China; 2 School of Sociality and Psychology, Southwest University for Nationalities, Chengdu, 610041 China; 3 Department of Radiology, Henan Provincial People’s Hospital & the People’s Hospital of Zhengzhou University, Zhengzhou, 450003 China; 4 Department of Psychology, School of Public Administration, Sichuan University, Chengdu, 610065 China; 5 Department of Psychology, Chengdu Mental Health Center, Chengdu, 610031 China

## Abstract

Achievement in school is crucial for students to be able to pursue successful careers and lead happy lives in the future. Although many psychological attributes have been found to be associated with academic performance, the neural substrates of academic performance remain largely unknown. Here, we investigated the relationship between brain structure and academic performance in a large sample of high school students via structural magnetic resonance imaging (S-MRI) using voxel-based morphometry (VBM) approach. The whole-brain regression analyses showed that higher academic performance was related to greater regional gray matter density (rGMD) of the left dorsolateral prefrontal cortex (DLPFC), which is considered a neural center at the intersection of cognitive and non-cognitive functions. Furthermore, mediation analyses suggested that general intelligence partially mediated the impact of the left DLPFC density on academic performance. These results persisted even after adjusting for the effect of family socioeconomic status (SES). In short, our findings reveal a potential neuroanatomical marker for academic performance and highlight the role of general intelligence in explaining the relationship between brain structure and academic performance.

## Introduction

Academic performance at the end of high school plays a crucial role in students’ future academics and career development. For instance, in China, the score of the Chinese National College Entrance Examination (CNCEE) (also known as Gaokao) taken at the end of high school is the sole criterion for admission to Chinese universities. Success in this examination offers not only a key opportunity for students to acquire subsequent academic and vocational achievement but also represents a critical promising opportunity for poverty-stricken families to change their fortunes^1, 2^. Therefore, exploring the factors related to academic performance in adolescents at the end of high school might be critical for possible reforms in education and curriculum.

Evidence from numerous studies has showed that a myriad of psychosocial factors contribute to academic performance^3^, which is usually measured by standardized tests (e.g., the Achievement College Test and the Stanford Achievement Test) or Grade Point Average (GPA)^4, 5^. Among these factors, general intelligence is the most stable and powerful predictor of academic performance^6, 7^. The mean correlation between general intelligence and academic performance is approximately 0.5^8, 9^, which varies considerably depending on the variability of the measures and samples. Furthermore, several studies have shown that general intelligence plays a causal role in academic performance^10–13^. In this research, we sought to explore the neuroanatomical correlates of academic performance and the role of general intelligence in the association between brain anatomy and academic performance by performing structural magnetic resonance imaging (S-MRI).

Although academic performance is a popular research topic in the fields of psychology and education^14, 15^, the association between academic performance and the brain remains largely unknown. Neuroscientific researchers have recently begun to examine the neurobiological basis underlying measures of academic performance. An electroencephalography study first reported that, during working memory tasks, high school students with higher academic performances exhibited greater frontal energy in the theta and delta frequencies^16^. Furthermore, as one of the most important facilities for psychoradiology (https://radiopaedia.org/articles/psychoradiology) ^17, 18^, Magnetic Resonance Imaging (MRI) has been widely used to investigate the human brain. Using a longitudinal functional MRI (fMRI) study design, Horowitz-Kraus *et al*. (2015) found that students’ college preparedness test scores can be predicted by the activities in the prefrontal cortex (PFC) (e.g., the dorsolateral prefrontal cortex [DLPFC] and the anterior cingulate cortex [ACC]) while performing narrative-comprehension tasks at 5–7 years of age^19^. In addition, a recent voxel-based morphometry (VBM) study based on region of interest (ROI) analysis reported a positive association between academic performance and regional gray matter volume (rGMV) of the frontal lobe in adolescents and children aged 4 to 22 years^20^. In summary, these findings consistently show that the PFC, which is known to be essential for complex learning, emotion regulation and inhibition control^21, 22^, may predict individual differences in academic performance.

The PFC is also commonly regarded as the neural center of individual differences in general intelligence^23–25^. First, many investigations of individuals with deficits in intelligent behavior and cognitive function caused by brain lesions have reported that the functioning of PFC regions plays an essential role in general intelligence^26–29^. Moreover, in healthy subjects, general intelligence has also been found to be associated with the functional and structural variations in PFC regions, which include the DLPFC, the ventrolateral PFC (VLPFC), the medial PFC (MPFC), the ACC and the supplementary motor area (SMA) (see a meta-analysis)^30^. Given the causal role of general intelligence in academic performance and the relationships between the PFC regions and academic performance, general intelligence might mediate the influence of the PFC on academic performance.

To carry out our investigations, we used the real-world academic performance, the standard measurement of general intelligence and the methodology of VBM. VBM analysis is one of the most popular and valid methods for evaluating the amount of gray matter in different brain areas^31^. Due to its low-cost and task-free condition characteristics, VBM analysis has been popularly used to investigate the brain correlates of the human mind and behavior^32, 33^. Considering previously reported brain findings regarding academic performance, we speculated the structural variations in PFC regions might predict individual differences in academic performance. Specially, to ensure adequate statistical power for a whole-brain analysis, we investigated the association of academic performance with brain structures in a large sample of adolescent students (N = 214) within a narrow age range because it is hard to identify individual differences across a broad age range^34^. In addition, in light of the causal role of general intelligence in academic performance and the relationships between PFC regions and general intelligence, we further hypothesized that general intelligence might play a mediating role in the associations of the structural variations in the PFC regions with academic performance. Finally, we tested the specificity of our findings by excluding the effects of family socioeconomic status (SES).

## Results

### Brain structure of academic performance

Table 1 presents the descriptive statistics of the measures, including the mean, standard deviation, range, skewness, and kurtosis. As is customary^35^, the scores for the measures were normally distributed because the skewness and kurtosis values ranged from −0.51 to 0.11, with the exception of age. Age was not correlated with CNCEE score (r = −0.04, *p* = 0.572). Male students demonstrated higher CNCEE scores than female students [t (212) = 4.10, *p* < 0.001]. After controlling for gender and age, we found that the total gray matter volume (GMV) was significantly correlated with CNCEE score (r = 0.21, *p* = 0.003). Next, we examined the neural substrates underlying academic performance.

**Table 1:** Descriptive statistics of participant-level variables.

Variable	Mean	SD	Range	Skewness	Kurtosis
Age	18.49	0.55	16–20	0.49	1.68
Total GMV	0.67	0.06	0.53–0.82	0.10	−0.51
CNCEE	521.32	70.13	301–645	−0.46	0.06
Term examination	544.38	45.61	401–633	−0.51	0.11
RAPM	24.16	5.64	6–36	−0.28	0.01
Family SES	5.29	1.49	1–9	−0.14	−0.15

*Note:* SD = standard deviation; GMV = gray matter volume; CNCEE = Chinese National College Entrance Examination; RAPM = Raven’s Advanced Progressive Matrix; SES = socioeconomic status. The values of term examination were based on 129 participants, other values were based on 214 participants.

**Figure 1:**
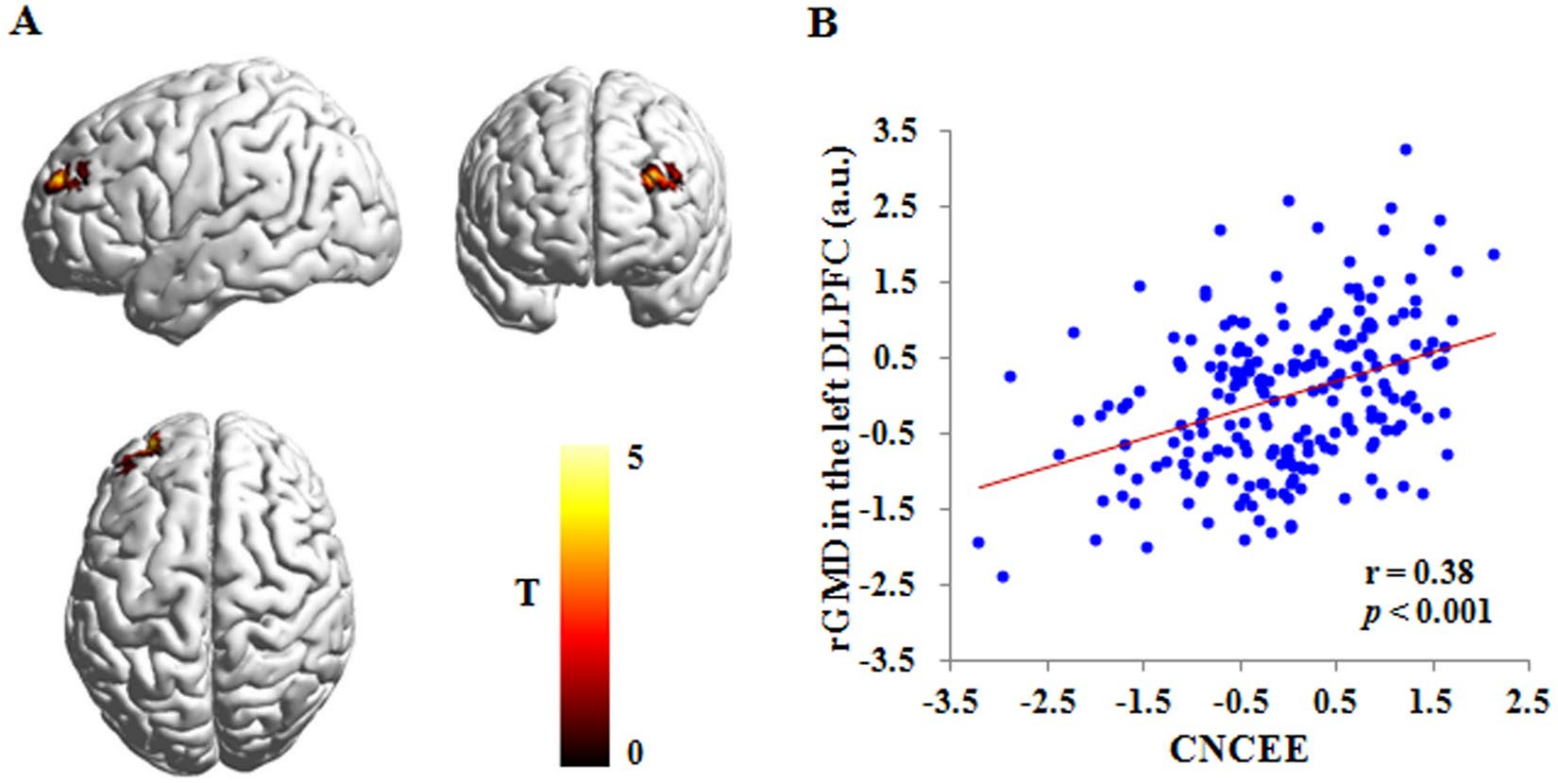
Brain regions that are related to CNCEE. (**A**) Brain images showing the positive association between the rGMD of the left DLPFC and CNCEE. (**B**) Scatter plot depicting the correlation between CNCEE and the rGMD of the left DLPFC (r = 0.38, *p* < 0.001). The score of horizontal axis represents the standardized residual of the CNCEE score (average standardized score of four curriculum subjects) after adjusting for age, gender and total gray matter volume. The score of vertical axis represents the standardized residual of the rGMD in the left DLPFC after adjusting for age, gender and total gray matter volume. The color bar represents the Student’s T-test (T) scores in each voxel, 0 represents the minimum T score and 5 represents the maximum T score. rGMD = regional gray matter density; DLPFC = dorsolateral prefrontal cortex; a.u. = arbitrary unit; CNCEE = Chinese National College Entrance Examination.

To identify the brain areas associated with academic performance, a whole-brain multiple regression analysis was conducted between individuals’ CNCEE scores and their regional gray matter density (rGMD) values in each voxel. The results revealed that CNCEE score was positively related to the rGMD of the left DLPFC (the middle frontal gyrus) (r = 0.38, *p* < 0.001) (see Table 2 and Fig. 1), after controlling for gender, age and the total GMV. We observed no other significant results. To examine the stability of the association of brain structure with academic performance, we first extracted the mean rGMD values of the region identified from the whole-brain analyses and then implemented a machine learning approach with four-fold balanced cross-validation procedures. The results showed that the rGMD of the left DLPFC can reliably predict individuals’ CNCEE scores [r_(predicted, observed)_ = 0.35, *p* < 0.001], after adjusting for gender, age and the total GMV.

**Table 2:** Brain regions where gray matter density was associated with academic performance.

Region	BA	Peak MNI coordinate			Peak T score	Cluster size (mm^3^)
		x	y	z		
Correlation with CNCEE						
Left DLPFC	9/10	−30	58	28	4.99	1552
Correlation with term examination						
Left DLPFC	9/10	−30	58	26	5.04	2224

*Note:* DLPFC = dorsolateral prefrontal cortex; BA = Brodmann’s area; MNI = Montreal Neurological Institute; CNCEE = Chinese National College Entrance Examination. We set the threshold for significant regions to p < 0.05 at the cluster level with an underlying p < 0.001 at the voxel level (non-stationary cluster correction).

**Figure 2:**
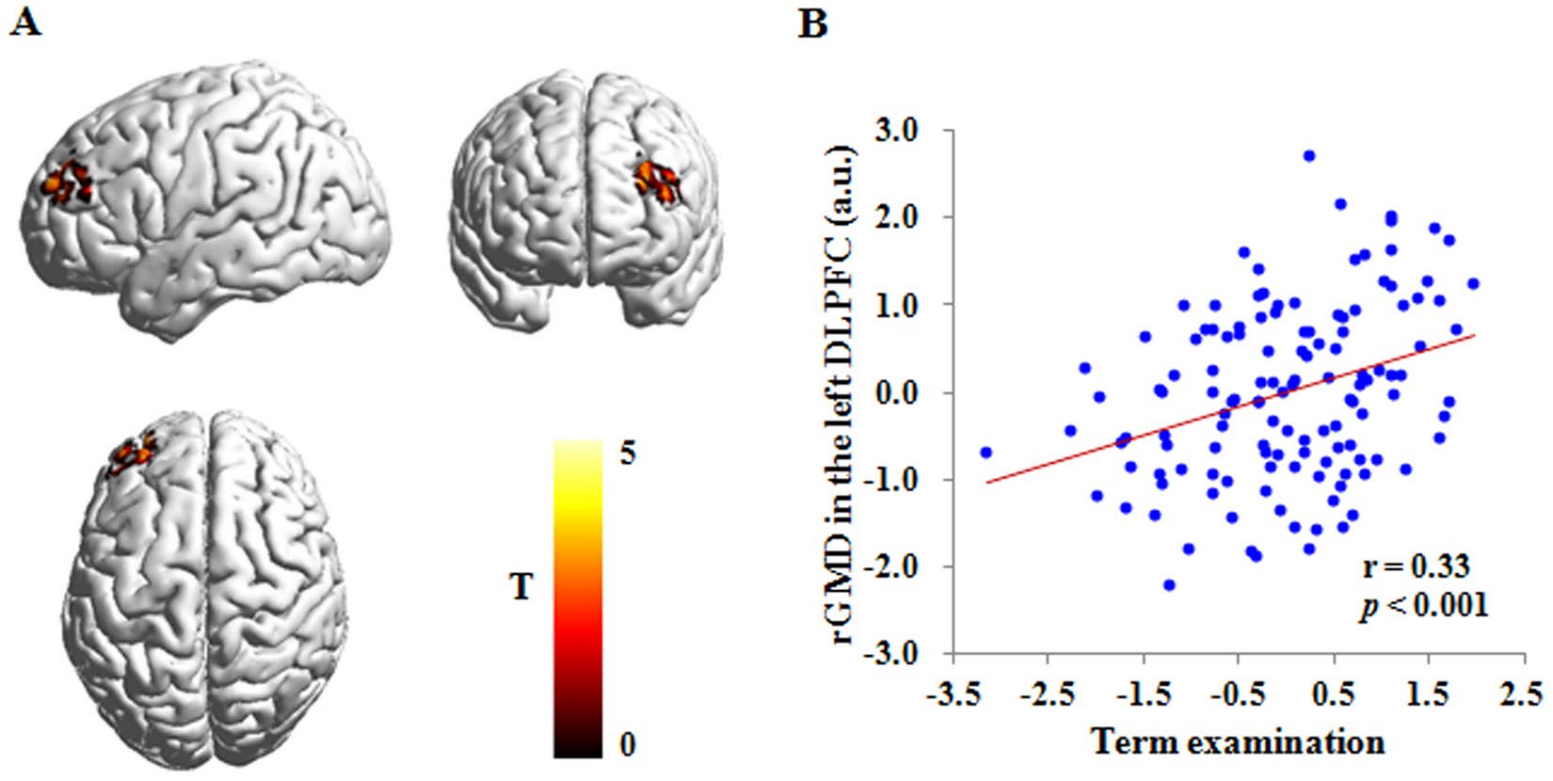
Brain regions that are related to term examination. (**A**) Brain images showing the positive association between the rGMD of the left DLPFC and term examination. (**B**) Scatter plot depicting the correlation between term examination and the rGMD of the left DLPFC (r = 0.33, *p* < 0.001). The score of horizontal axis represents the standardized residual of the term examination score (average standardized score of nine curriculum subjects) after adjusting for age, gender and total gray matter volume. The score of vertical axis represents the standardized residual of the rGMD in the left DLPFC after adjusting for age, gender and total gray matter volume. The color bar represents the Student’s T-test (T) scores in each voxel, 0 represents the minimum T score and 5 represents the maximum T score. rGMD = regional gray matter density; DLPFC = dorsolateral prefrontal cortex; a.u. = arbitrary unit.

To test the stability of the academic performance across time, we collected term examination score in part of our participants (129 students). Behaviorally, this term examination score was highly correlated with CNCEE score (r = 0.71, *p* < 0.001). Neurally, the rGMD in the left DLPFC related to CNCEE score could significantly predict term examination score (r = 0.38, *p* < 0.001). More importantly, whole-brain regression analyses showed that term examination score was related to the rGMD in the same region (the left DLPFC; r = 0.33, *p* < 0.001; see Table 2 and Fig. 2), after adjusting for gender, age and the total GMV. We observed no other significant results in the whole-brain regression analyses. Then, we performed a prediction analysis to test the stability of the association between brain structure and term examination score. The results showed that the rGMD of the left DLPFC can reliably predict individuals’ term examination scores [r_(predicted, observed)_ = 0.30, *p* < 0.001], after adjusting for gender, age and the total GMV. Considering the high correlation between CNCEE score and term examination score and the same brain region observed between CNCEE score and term examination score, we used only CNCEE score as the measure of academic performance in subsequent analyses.

**Figure 3:**
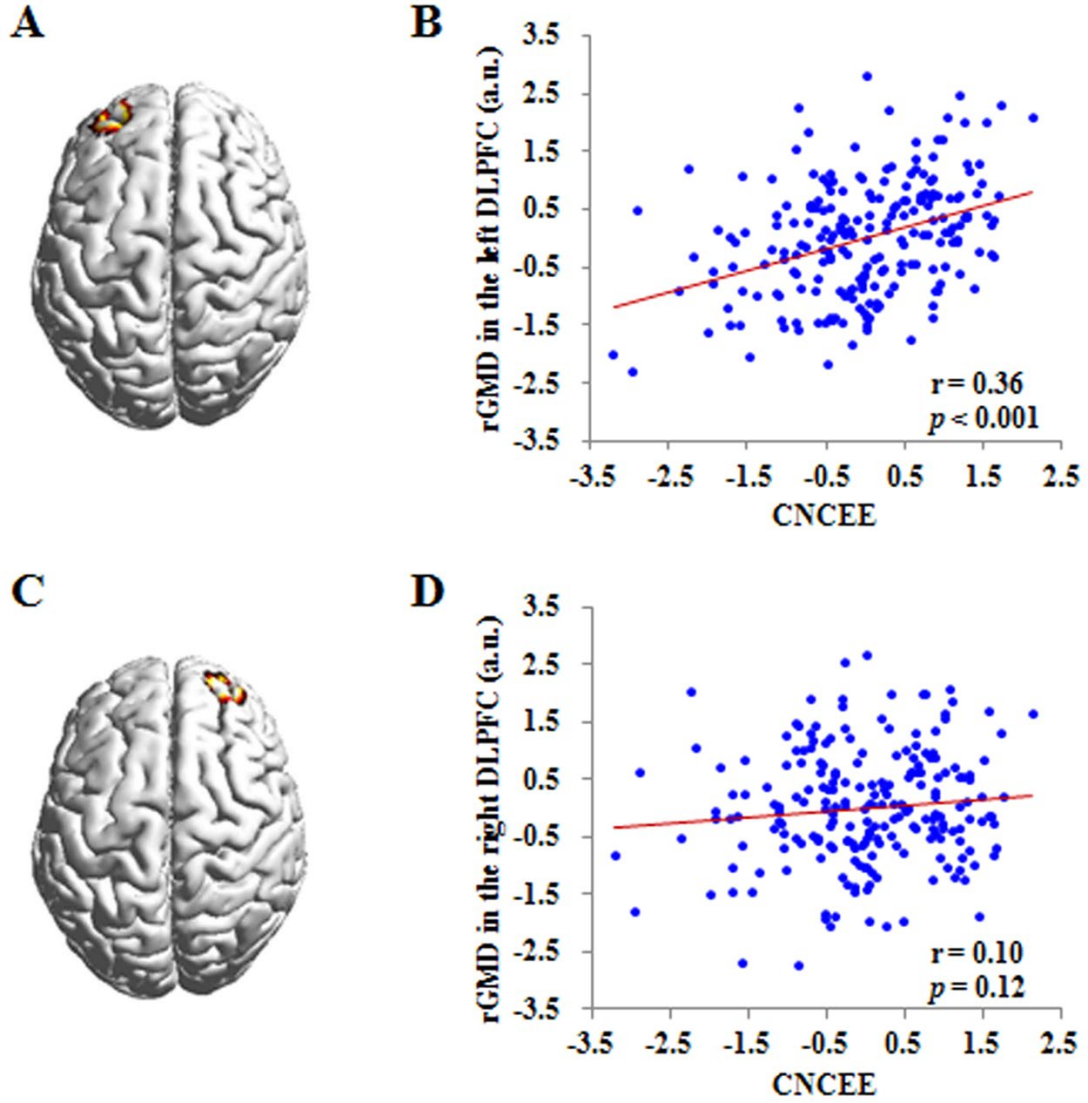
Correlation between CNCEE and the rGMD in the left DLPFC and right DLPFC. (**A**) Brain image showing the spherical region of interest (ROI, radius = 10 mm) in the left DLPFC, which was created by using the coordinate of peak (−30, 58, 28) in the significant region related to CNCEE from the whole-brain regression analyses. (**B**) Scatter plot depicting the correlation between CNCEE and the rGMD of the left DLPFC (r = 0.36, *p* < 0.001). The score of horizontal axis represents the standardized residual of the CNCEE score (average standardized score of four curriculum subjects) after adjusting for age, gender and total gray matter volume. The score of vertical axis represents the standardized residual of the rGMD in the left DLPFC after adjusting for age, gender and total gray matter volume. (**C**) Brain image showing the spherical ROI (radius = 10 mm) in the right DLPFC, which was created by using the coordinate of peak (30, 58, 28) in contrast to the left DLPFC. (**D**) Scatter plot depicting the correlation between CNCEE and the rGMD of the right DLPFC (r = 0.10, *p* = 0.12). The score of horizontal axis represents the standardized residual of the CNCEE score (average standardized score of four curriculum subjects) after adjusting for age, gender and total gray matter volume. The score of vertical axis represents the standardized residual of the rGMD in the right DLPFC after adjusting for age, gender and total gray matter volume. rGMD = regional gray matter density; DLPFC = dorsolateral prefrontal cortex; a.u. = arbitrary unit; CNCEE = Chinese National College Entrance Examination.

Finally, to confirm the specificity of the association between academic performance and left DLPFC density, we first created a spherical ROI (radius = 10 mm) in the left DLPFC by using the coordinate of peak (−30, 58, 28) in the significant region associated with CNCEE score that was detected from the whole-brain regression analyses. Correspondingly, we created another spherical ROI (radius = 10 mm) in the right DLPFC by using the coordinate of peak (30, 58, 28). Then, we extracted the mean rGMD values in these ROIs and explored their associations with academic performance. As depicted in Fig. 3, CNCEE score was correlated with the rGMD of the left DLPFC (r = 0.36, *p* < 0.001) but not with the rGMD of the right DLPFC (r = 0.10, *p* = 0.12).

### The relationship between brain structure and academic performance was mediated by general intelligence

To explore the role of general intelligence in the relation of brain structure and academic performance, we collected Raven’s Advanced Progressive Matrix (RAPM) data in our sample. First, we confirmed a significant correlation between general intelligence and CNCEE score (in our dataset: r = 0.38, *p* < 0.001). Moreover, general intelligence explained additional variance in CNCEE score (*ΔR* ^2^ = 8.7%, *p* < 0.001) beyond that explained by gender, age and the total GMV. Second, we tested whether the rGMD of the left DLPFC that was related to CNCEE score could predict general intelligence. The results revealed that the rGMD of the left DLPFC was significantly related to general intelligence (r = 0.34, p < 0.001). Furthermore, the left DLPFC density explained additional variance in general intelligence (*ΔR* ^2^ = 3.2%, *p* < 0.001) beyond that explained by gender, age and the total GMV.

**Figure 4:**
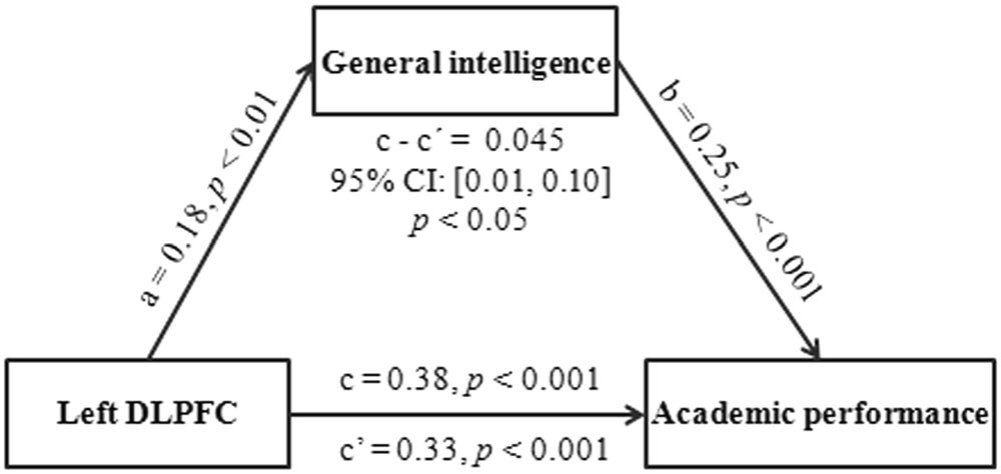
General intelligence mediates the relationship between the left DLPFC and academic performance. The panel shows that the left DLPFC affects academic performance through general intelligence. Here, we used the standardized regression coefficients to indicate the paths between variables, and all paths (a, b, c and c’) were significant. The indirect effect (c − c’ or a × b) was significant. Academic performance was measured using the Chinese National College Entrance Examination (CNCEE) score. Gender, age and the total gray matter volume were controlled for. DLPFC = dorsolateral prefrontal cortex.

These results suggested that there were close relationships among brain anatomy, academic performance and general intelligence, although the exact associations among them remain unknown. To investigate whether general intelligence could mediate the relation of brain structure and academic performance, we carried out mediation analyses with gender, age and the total GMV as controlling variables. We found that, after including general intelligence as an intermediate variable, the association of the rGMD of the left DLPFC with CNCEE score reduced, although it was still significant (see Fig. 4). The 5,000 bootstrap simulations further demonstrated that general intelligence mediated the influence of the left DLPFC on CNCEE score (indirect effect = 0.045, 95% CI = [0.01, 0.10], *p* < 0.05).

Finally, we included family SES as a covariate to test the specificity of our findings. First, we checked the impact of SES on the neural correlates of academic performance. After adjusting for SES, CNCEE score was still related to the rGMD of the left DLPFC (r = 0.38, *p* < 0.001). Second, we investigated the influence of SES on the association of brain structure with general intelligence. The results demonstrated that the rGMD of the left DLPFC was still associated with general intelligence (r = 0.17, *p* = 0.01) even after adjusting for SES. Third, we examined whether SES affected the indirect effect of general intelligence on the relationship between brain structure and academic performance. The results revealed that, after controlling for SES, general intelligence still accounted for the common variance between the rGMD of the left DLPFC and CNCEE score (indirect effect = 0.043, 95% CI = [0.01, 0.09], *p* < 0.05). Gender, age and the total GMV were controlled for in these analyses. In summary, these results suggested that our findings were not affected by family SES.

## Discussion

The current study investigated the association between academic performance and brain structure and the role of general intelligence in this association in a sample of healthy high school students. We observed two main findings. First, whole-brain regression analyses revealed that higher academic performance was correlated with greater rGMD of the left DLPFC. Second, mediation analyses demonstrated that general intelligence mediated the influence of the rGMD of the left DLPFC on academic performance. Our results persisted even after adjusting for the effect of family SES, indicating the specificity of these effects. According to our knowledge, this is the first investigation to directly examine the relationship between academic performance and rGMD in adolescent students. In brief, our study provides initial evidence for the neuroanatomical correlates of academic performance and reveals that general intelligence plays a mediating role in the association between brain structure and academic performance.

Confirming our first hypothesis, we observed that academic performance was significantly related to the rGMD of the left DLPFC. This positive association was consistent with prior studies that reported relationships between the structure and function of the DLPFC and measures of academic performance^16, 20^. Indeed, the DLPFC has been consistently considered as a neural hub related to psychological attributes in the intersection of cognitive and non-cognitive capacities^21, 36, 37^. On one hand, many prior studies have reported that the DLPFC is involved in a variety of cognitive capacities such as executive function^38, 39^, working memory^40, 41^, top-down attention^42, 43^, and reasoning and problem-solving^44, 45^. On the other hand, the DLPFC has also been linked with many non-cognitive capacities such as planning^46, 47^, emotional regulation^48, 49^, personality trait of conscientiousness^50^, self-regulation^51^, and self-esteem^52^. Behaviorally, it has also been revealed that academic performance apparently relies on these cognitive and non-cognitive factors^3, 53–56^. Therefore, the rGMD of the left DLPFC might be associated with academic performance through its relation to the cognitive and non-cognitive functions reviewed above.

In addition, our study showed that left DLPFC but not right DLPFC can predict individual differences in academic performance. One possible explanation for this result is that the measures of academic performance used in this study (i.e., CNCEE score and term examination score) mainly required participants’ language ability compared with non-language ability. This feature of the CNCEE score and term examination score may cause the result of the significant association between academic performance and the rGMD of the left DLPFC, which partly overlapped with the Broca’s area, a well known brain region for language processing in the left PFC^57^. Moreover, the present study only observed significant association with the left DLPFC but not with the other brain regions (e.g., temporal lobe and occipital lobe), which are found to be critical for measures of academic performance^20, 34^. Thus, future studies may consider using other measures of academic performance (e.g., GPA) to examine the brain basis of academic performance.

Confirming our second hypothesis, the influence of the rGMD of the left DLPFC on academic performance was mediated by general intelligence. Behaviorally, the stable relationship between general intelligence and academic performance has been repeatedly reported in previous investigations^6–13^, which fit well with the correlation observed in our dataset (r = 0.38, *p* < 0.001). Moreover, some investigators even questioned whether other psychological factors could account for additional variance in academic performance beyond the variance accounted for by general intelligence because the influence of general intelligence is so strong^58^. Therefore, general intelligence is likely a dominant factor in shaping the academic performance of individuals. In addition, the present study revealed a positive association of DLPFC density with general intelligence, which well corroborates previous findings that revealed relationships between the structure and function of the DLPFC and general intelligence in healthy individuals (see a meta-analysis)^30^. The literature reviewed above has shown that the DLPFC is implicated in many higher-order cognitive functions including executive function, working memory, top-down attention, and reasoning and problem-solving^38–45^. These cognitive functions have also been verified to be crucial for performing well on standard intelligence tests^59–62^. Taken together, our findings highlight that general intelligence might act as an underlying mechanism for explaining the relation between the structural variations in the DLPFC and academic performance.

The present study has several limitations that deserve consideration in future studies. First, we could not determine the direction of causality regarding the relationships among academic performance, general intelligence and brain structure because of the cross-sectional design used in this study. Future investigators might consider using longitudinal designs to examine the causal direction of these associations. Second, the participants included in this study were a group of high school students with a narrow age range. Therefore, future research could extend upon our study to include more diverse populations, such as primary school students and undergraduates. Third, because we used only the rGMD as the measure of brain structure, future researchers may consider employing other measures of brain structure and function to investigate the neural basis of academic performance. In particular, functional activations during task conditions (i.e., task-based fMRI) and task-free conditions (i.e., resting-state fMRI) are different from the characteristics of brain structure. It is well known that brain regions work as a functional network in the processing of corresponding psychological ability^63^. Given academic performance consists of various complex cognitive and non-cognitive abilities, future studies are encouraged to explore the associations between academic performance and brain networks by using task-based fMRI and resting-state fMRI.

In conclusion, we explored the brain correlates of academic performance using a VBM approach with S-MRI. We found that higher academic performance was related to greater rGMD of the left DLPFC, which sheds light on the underlying brain basis that determines academic performance. Furthermore, general intelligence mediated the impact of DLPFC density on academic performance, revealing a potential mechanism that may explain the common variance between brain structure and academic performance. These results remained significant even after adjusting for family SES, suggesting the robustness of these effects. Overall, our findings present the first evidence that rGMD serves as the neural basis of academic performance and reveal the role of general intelligence in the association between brain structure and academic performance.

## Methods

### Participants

Two hundred thirty-four healthy adolescent students who reported no history of psychiatric or neurological illness were recruited to participate in this study. However, twenty students with no behavioral testing scores or abnormal brain structures were excluded. Thus, a total of two hundred fourteen students (114 females; M_age_ = 18.49 years, SD = 0.55) were included in the data analyses. We recruited the participants from an ongoing project with primary goals of investigating the determinants of academic achievement, well-being and social cognition among adolescents in Chengdu, China. All participants were right-handed according to the Edinburgh Handedness Inventory^64^ and had recently graduated in June 2015 from several local public high schools. Experiments were conducted from June 2015 to September 2015, and each participant provided written informed consent before testing. This study was approved by the local research ethics committee of the West China Hospital of Sichuan University. The study protocols were performed in accordance with the approved guidelines and regulations.

### Behavioral measures

#### Academic performance

We used the average standardized scores of CNCEE, which consists of tests of four curriculum subjects (Chinese, English, Mathematics and Comprehensive Ability), to measure participants’ academic performances. The scores of the CNCEE range from 0 to 750, which comprehensively reflect the learning processes and outcomes during three years of high school in Chinese students. The participants took the CNCEE on June 7 and June 8, 2015. We obtained the CNCEE scores from the Chengdu Education Institute database.

To ensure the stability of the academic performance across time, we collected the data of a term examination in a subsample of our participants (73 females, 56 males; Mage = 18.45 years, SD = 0.54), which are part of our larger longitudinal project. This unified term examination consists of tests of nine curriculum subjects, including Chinese, English, Mathematics, Physics, Chemistry, Biology, Politics, History, and Geography. The participants took this term examination on June 2014 to monitor learning progress. We used the average standardized scores of the nine curriculum subjects as another measure of academic performance in our dataset. These scores have been used in previous study^65^.

#### General intelligence

To assess individuals’ general intelligence, the RAPM^66^, which is one of the most popular and sound instruments for evaluating intelligence, was administered. This measure includes 36 non-verbal items that refer to abstract reasoning. During testing, participants were instructed to choose the missing figure for each graphical matrix within 30 minutes^67^. The participants completed RAPM and several questionnaires after their MRI scans. All of these tests were completed in a quiet room at the West China Hospital of Sichuan University. Two research assistants who were blind to this study supervised these testing sessions, and schoolteachers were not present. Therefore, the participants were confident about the confidentiality of their responses. RAPM scores were obtained by summing the number of correct answers, with higher scores representing higher levels of general intelligence. In our sample, this test exhibited an adequate internal reliability (Cronbach’s α = 0.82).

#### Family SES

To rule out the potential influence of family SES on the association between academic performance and brain structure^34, 68–70^, we employed a one-item scale as a graph of a ladder with ten rungs^71^. The participants were asked to rank the levels of their parents’ occupational prestige, income and education with ranging from 1 (lowest rank) to 10 (highest rank). Compared to objective SES, prior evidence has suggested that subjective SES exhibits a greater association with health-related measures^71^.

### MRI data acquisition and preprocessing

#### Data acquisition

MRI experiments were performed using a Germany Siemens-Trio Erlangen 3.0 T MRI scanner located at the West China Hospital of Sichuan University and equipped with a 12-channel head coil. We used a magnetization-prepared rapid gradient echo sequence to obtain the T1-weighted anatomical images with the following scanning parameters: 176 slices; slice thickness, 1 mm; flip angle, 9°; matrix size, 256 × 256; repetition time, 1900 ms; inversion time, 900 ms; echo time, 2.26 ms; voxel size, 1 mm × 1 mm × 1 mm.

#### Data preprocessing

We preprocessed the MRI data with the Statistical Parametric Mapping program (SPM8, Wellcome Department of Cognitive Neurology, London, UK). First, a medical radiologist who was blinded to the study design visually inspected each image. Three participants were excluded because of the abnormal brain structures (e.g., unusual cyst). Second, we manually set the origin of the images to the anterior commissure for better registration. Third, we used the new segmentation in SPM8 to segment the images into white matter and gray matter. Next, we conducted registration, normalization and smoothness analyses by using Diffeomorphic Anatomical Registration Through Exponentiated Lie algebra (DARTEL) in SPM8^72^. To do so, we aligned and resampled the gray matter images to 2 mm × 2 mm × 2 mm and then normalized them to a study specific template in the MNI152 space. Finally, we applied an 8-mm full-width at half-maximum Gaussian kernel to smooth the normalized gray matter images. The resulting images, which represent the rGMD, were used in the following analyses.

Here, we used rGMD but not rGMV as the index of gray matter. While rGMV reflects the absolute volume of the gray matter, rGMD reflects the relative concentration of the gray matter. Although the implications of the differences between rGMD and rGMV are not well known, both have been widely employed in previous structural investigations, and there is a high similarity in the results observed based on the two parameters^31, 73^. However, rGMD has been more frequently used in developmental studies than rGMV because the gray matter in brain areas such as the PFC is thinning during normal development^74–76^. Given that the participants of the current study were adolescents, which is considered as a critical period for cortical thinning^77, 78^, we selected rGMD as the gray matter index.

### Statistical analyses

#### VBM analysis

To examine the associations of brain structures with academic performance, we conducted a whole-brain multiple regression analysis with the rGMD in each voxel as the dependent variable, the CNCEE score as the independent variable, and gender, age and total GMV as confounding variables. Moreover, we conducted another whole-brain multiple regression analysis with the rGMD in each voxel as the dependent variable, the term examination score as the independent variable, and gender, age and total GMV as confounding variables. In these analyses, we applied an absolute threshold mask of 0.2 to remove the edge effects between the white matter and gray matter. To determine the regions of significance, we used non-stationary cluster correction based on the random field theory^79^. Specifically, we set the cluster-level threshold at *p* < 0.05 combined with the underlying voxel-level at *p* < 0.001. The non-isotropic cluster-size test has been widely employed in prior studies that have analyzed VBM data^80–82^. We performed these analyses using SPM8 software.

#### Prediction analysis

We employed a machine learning approach to investigate the stability of the association between brain structure and academic performance. This approach is based on balanced cross-validation employing linear regression^80, 83, 84^. In the analysis, we input academic performance as the dependent variable and the rGMD of the region(s) as the independent variable. The predictive ability of the independent variable on the dependent variable was measured by r_(predicted, observed)_, which was evaluated using a four-fold balanced cross-validation procedure. First, we divided the data into four folds to ensure that the distributions of the independent variable and the dependent variable across the folds were balanced. Second, we used three folds to build a linear regression model, with the fourth fold left out. Then, we employed the model to predict the data of the fourth fold. We repeated this procedure four times to calculate a final r_(predicted, observed)_, which represents the association between the observed data and the data predicted by the regression model. Here, we employed a nonparametric testing method to determine the statistical significance of the model. Specially, we generated 1000 surrogate datasets to estimate the null distribution of r_(predicted, observed)_, where the null hypothesis corresponds to no relationship between academic performance and rGMD. Then, we permuted the labels of the observed data points to generate each surrogate dataset D_i_ of size equal to the observed dataset. Next, we used the predicted labels with the four-fold balanced cross-validation procedure and the actual labels of D_i_ to calculate the r_(predicted, observed)_ of D_i_ [i.e., r_(predicted, observed)i_]. Finally, we counted the number of r_(predicted, observed)i_ values greater than r_(predicted, observed)_ and then divided that count by the number of Di datasets (1000). The resulting value was treated as the statistical significance (*p* value).

#### Mediation analysis

To confirm whether general intelligence can mediate the impact of brain anatomy on academic performance, we performed mediation analyses with the SPSS macro program^85^. In the standard three-variable mediation model, we included academic performance as the dependent variable, brain anatomy as the independent variable and general intelligence as the mediator variable. According to the conventions^86^, path c is the association of the independent variable with the dependent variable (total effect), path c’ is the association of the independent variable with the dependent variable after adjusting for the mediator variable (direct effect), and the indirect effect is equal to path a (relation of the independent variable and the mediator variable) × path b (relation of the mediator variable and the dependent variable after controlling for the independent variable) or path c - path c’. The significance of the indirect effect was determined by using the bootstrapping procedure^85^. Here, we used 5,000 samplings to generate 95% confidence intervals (CIs). If a CI did not contain zero, then the association between the independent variable and the dependent variable was significantly explained by the mediator variable (*p* < 0.05).
